# Haplotype-resolved de novo assembly of the Vero cell line genome

**DOI:** 10.1038/s41541-021-00358-9

**Published:** 2021-08-20

**Authors:** Marie-Angélique Sène, Sascha Kiesslich, Haig Djambazian, Jiannis Ragoussis, Yu Xia, Amine A. Kamen

**Affiliations:** 1 Department of Bioengineering, McGill University, Montreal, QC Canada; 2 McGill Genome Center, Montréal, QC Canada

**Keywords:** Biotechnology, Diseases

## Abstract

The Vero cell line is the most used continuous cell line for viral vaccine manufacturing with more than 40 years of accumulated experience in the vaccine industry. Additionally, the Vero cell line has shown a high affinity for infection by MERS-CoV, SARS-CoV, and recently SARS-CoV-2, emerging as an important discovery and screening tool to support the global research and development efforts in this COVID-19 pandemic. However, the lack of a reference genome for the Vero cell line has limited our understanding of host–virus interactions underlying such affinity of the Vero cell towards key emerging pathogens, and more importantly our ability to redesign high-yield vaccine production processes using Vero genome editing. In this paper, we present an annotated highly contiguous 2.9 Gb assembly of the Vero cell genome. In addition, several viral genome insertions, including Adeno-associated virus serotypes 3, 4, 7, and 8, have been identified, giving valuable insights into quality control considerations for cell-based vaccine production systems. Variant calling revealed that, in addition to interferon, chemokines, and caspases-related genes lost their functions. Surprisingly, the ACE2 gene, which was previously identified as the host cell entry receptor for SARS-CoV and SARS-CoV-2, also lost function in the Vero genome due to structural variations.

## Introduction

Originated from a female *Chlorocebus sabaeus* (African Green Monkey) kidney, the Vero cell line represents the most widely used continuous cell line for the production of viral vaccines with over 40 years of experience^1^. This includes the development and production of vaccines against dengue fever, influenza, Japanese encephalitis, polio, rabies, rotavirus, smallpox and more recently, Ebola (using a recombinant vesicular stomatitis virus)^2–4^.

The advances in gene editing have made it possible to edit the genome of cell lines with high-throughput and cost-effective methods using available genomic data, thus providing new possibilities for cell line development and vaccine bioprocessing intensification. Some attempts to develop engineered Vero cell line have been made^5^ but genome editing of this cell line still suffers from the lack of annotated reference-grade genomic information. Despite the publication of the Vero genomic landscape^6^, there are no tools available yet to efficiently select CRISPR/Cas9 target sites with sufficient accuracy such as a CRISPR screening library for Vero cells.

Furthermore, Vero cells have been identified as the cell line with the highest susceptibility to MERS-CoV^7^, SARS-CoV, and recently SARS-CoV-2^8^. Consequently, Vero cells have been extensively used in the current response to COVID-19 as a platform for SARS-CoV-2 isolation and replication, viral vaccine production, and identification of potential drug targets^9^. Currently, several COVID-19 attenuated or inactivated vaccine candidates in preclinical and clinical trials use Vero cells as a production platform. We thus propose a haplotype resolved annotated assembly of the WHO-Vero genome which will provide a valuable resource for quality control, enable the generation of high-throughput engineered sub-cell lines, and accelerate the development of vaccine manufacturing platforms contributing to the global preparedness plan to counteract emerging and reemerging infectious diseases.

## Results

### De novo assembly of the Vero genome and annotation

**Fig. 1:**
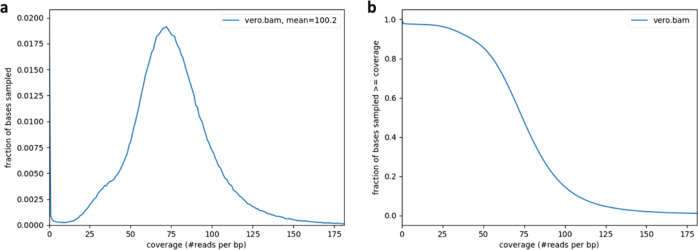
Vero genome sequencing depth. **a** Distribution of read coverages. **b** Genome fractions depth of sequencing.

**Fig. 2:**
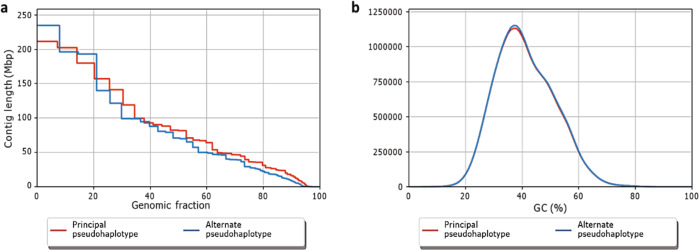
Quality control of the Vero genome de novo assembly. **a** Nx plot: Length ordered contigs. **b** Distribution of GC content in the contigs.

Using sequencing reads with a mean coverage per base pair of 100.2 (Fig. 1) of the African Green Monkey genome^10^, we present here a principal pseudohaplotype and an alternate pseudohaplotype of the Vero genome consisting respectively of 6872 and 6876 scaffolds, with a total length of 2.9 Gb, a L50 count of 12, and NG50 length of 82 and 70 Mb (Fig. 2) with 39,449 predicted genes (29,824 genes were predicted for the African Green Monkey genome using the same default parameters), 35,004 genes and pseudo genes annotated (including 21,620 protein-coding genes).

The completeness of the genome assembly was assessed by BUSCO^11^ and CEGMA^12^ via the gVolante portal^13^. 94.85% complete, 2.15 partial genes, and 94.85 complete, 2.57% partial genes were identified in the principal pseudohaplotype and the alternate pseudohaplotype, respectively. CEGMA^12^ revealed that, 98.71% of the 233 core vertebrate genes were evolutionarily conserved genes identified in both pseudohaplotypes of the Vero genome.

Comparing both pseudohaplotypes output before downstream processing, we find that from the initial 55,755 scaffolds 723 scaffolds have differing sequences. These 723 scaffolds account for 2,512,305,804 bases from a total of 2,848,013,978 assembled bases. In these differences, there are 5,027,642 mismatch (SNP) bases with 4,830,436 of 1 bp, 94,229 of 2–5 bp, and 217 of 6–25 bp. Comparing the pseudohaplotypes we also count indel of different sizes: 436,916 indels of 1 bp, 327,037 indels of 2–5 bp, and 103,216 indels of 6–25 bp.

Our assembly sequence quality was further confirmed by the 96.5% alignment of randomly generated illumine short reads downloaded from the SRA database. Furthermore, using those short reads, the QV (quality value) was calculated using Merqury^14^ pipelined with Meryl^15^ and pseudohaplotype 1 and 2 reached, respectively, a QV of 28.7531 and 28.3972 which correspond to an accuracy of 99.87 and 99.86%. Furthermore, a new 30X sequencing round was done and short reads were generated and aligned to the Vero genome for SNP call. Considering calls with AF of 1, an error rate of 0.015523% or one error every 6441 bases was observed.

Additionally, Vero RNAseq data were deposited under the Vero Bioproject alongside the two Vero genome pseudohaplotype assemblies in order to be used with vervet RNAseq data as evidence for annotation by NCBI. Comparing the resulting annotation (Annotation release 102) with the previous African Green Monkey annotation(Annotation release 100), only 1% of the genes in the Vero annotation are identical (i.e., Genes with perfect match in exon boundaries) to those of the African green monkey annotation, 46% of the genes had minor changes (i.e., Highly similar genes, with support scores of 0.66 or more (on a scale of 0 to 1) on both sides of the comparison, the support score is derived from a combination of matching exon boundaries and sequence overlap), 23% of the genes have major changes (i.e., Genes with support scores lower than 0.66 (on a scale of 0 to 1) on one or both sides of the comparison, and genes with changed locus, biotype or changed completeness, and split or moved genes), 30% of the genes are new (i.e., Novel genes or genes without a match in the African Green Monkey annotation). In addition, 68 viral proteins (36 viral genes) were also annotated.

### Detection of genomic rearrangements in the Vero cell line

**Fig. 3:**
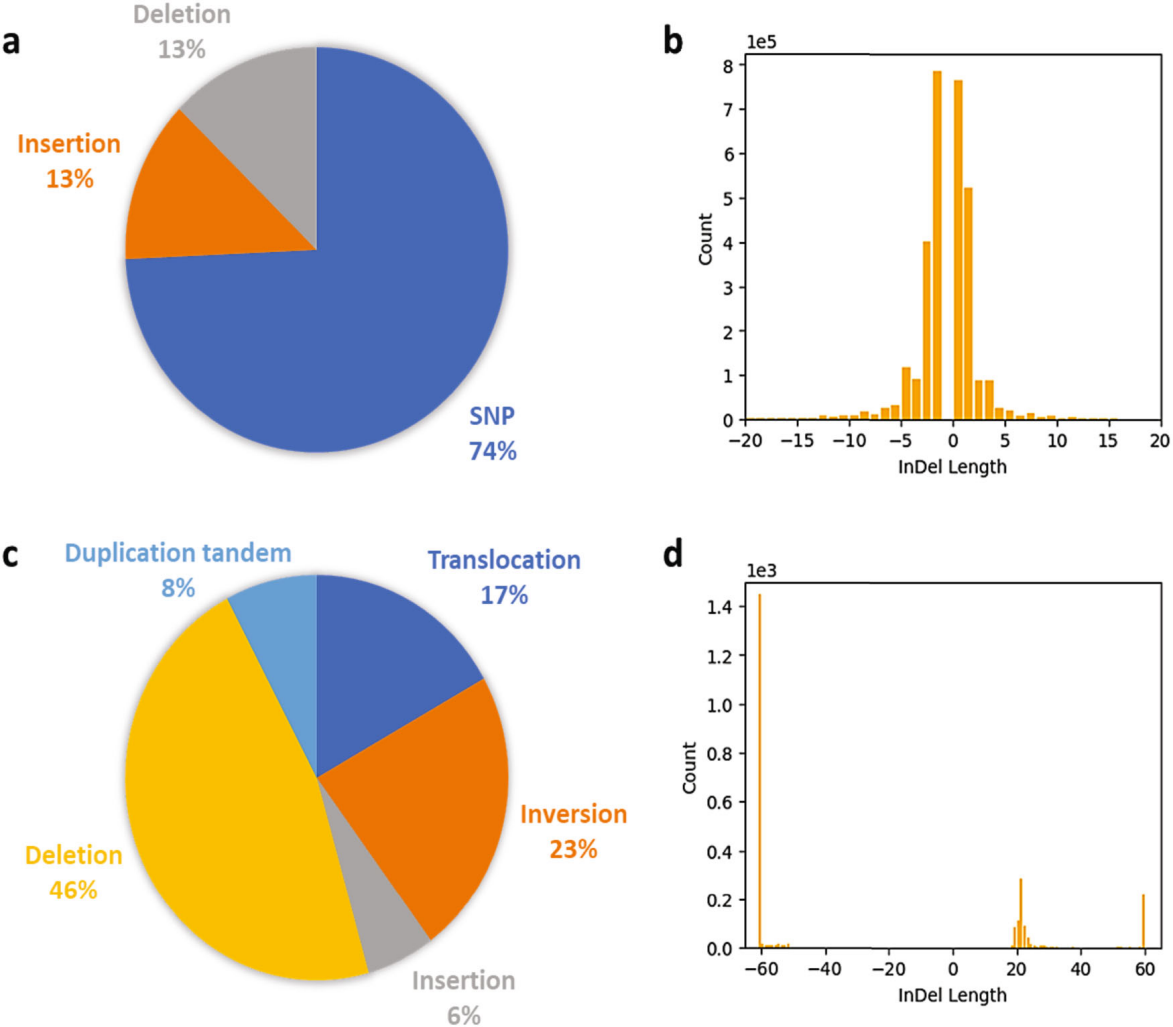
Characterization of called structural variants. **a** Different SNVSniffer called small-variant types distributions **b** SNVSniffer called small-scale indels distribution. **c** Different Manta called small-variant types distributions. **d** Manta called large-scale indels distribution.

**Fig. 4:**
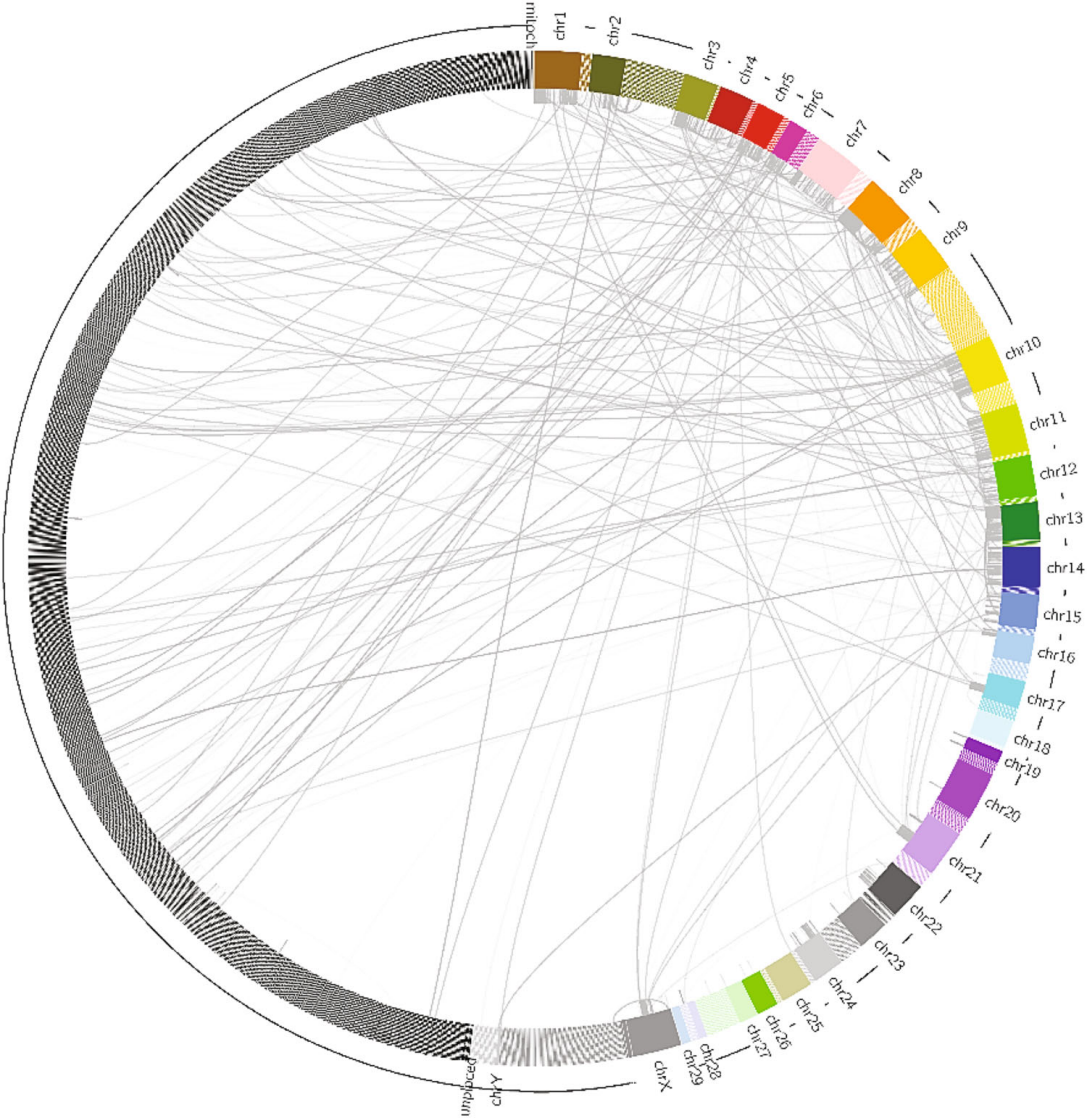
Circos plot of Manta called large-scale variants. The outer circle represents the African Green Monkey genome including its unplaced scaffolds (left dark gray) to highlight the interchromosomal translocations in the Vero genome relative to the African Green Monkey genome.

Using the African Green Monkey genome^10^ as a reference, the Vero cell line sequenced reads covered 91.3% of the African Green Monkey genome while ~12 million small indels and SNPs were called with SNVSniffer^16^ (Fig. 3a, b) and 7354 large-scale structural variants (including interchromosomal translocations) were called using Manta^17^ (Figs. 3c, d, and 4).

**Fig. 5:**
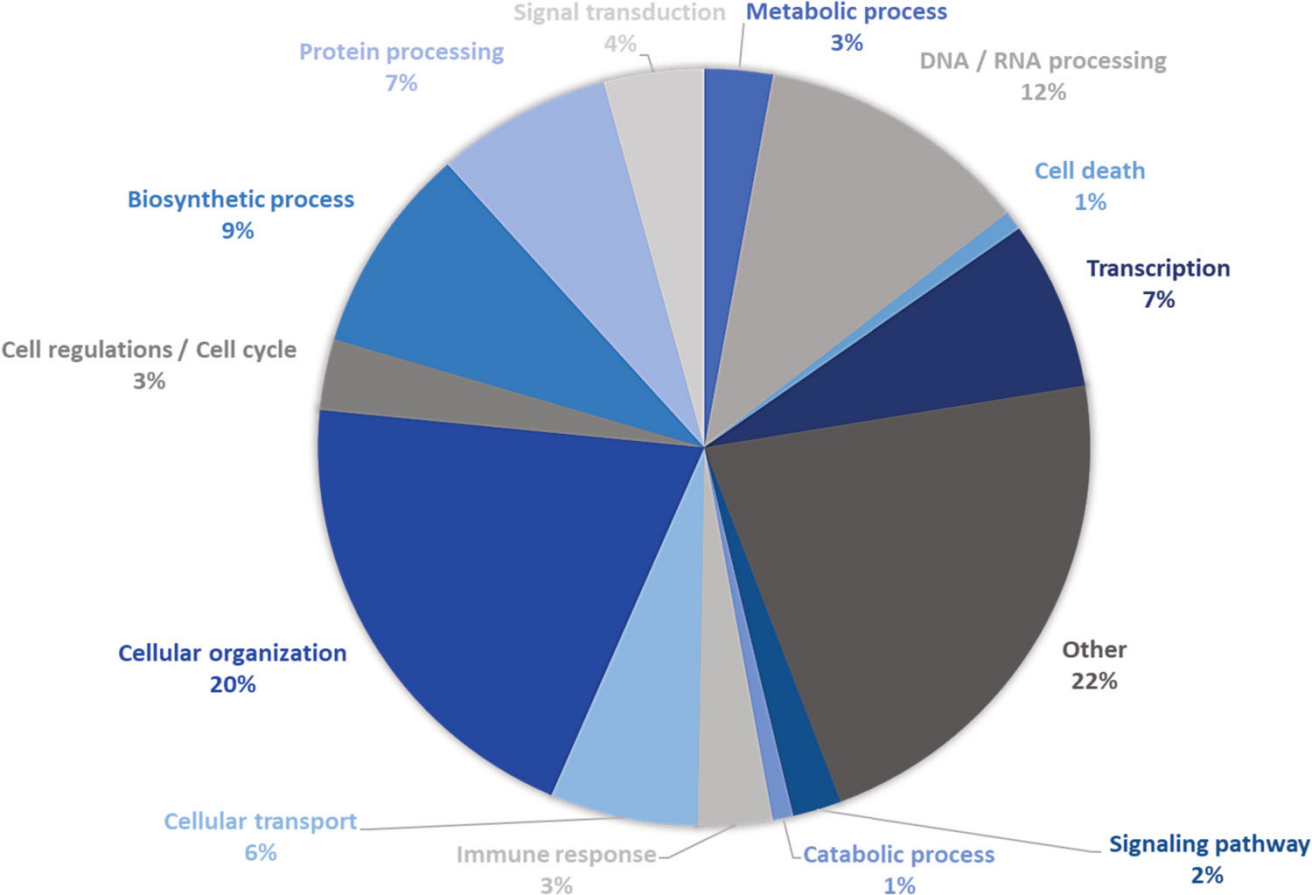
Function lost genes clustering. Biological clustering of genes identified as having lost their function based on their functional annotation.

Following the annotation of those variants, among 7585 genes predicted as having lost their function, a total of 551 genes were identified as having lost their function as a result of feature ablation (notably due to chromosome copy number variations), while 12 genes were identified as having lost their function due to transcript ablation (Table 1 and Supplementary Datas1–3). Besides these ablation variants, given the nature of the loss of function predictions, additional analysis such as proteomics might be needed in order to provide a detailed insight on the effect of those variants-caused gene loss of function on gene products functionality for instance in cases such as ACE2 or IVNS1AB predicted loss of function. After filtering, the 7585 genes with a predicted loss of function are involved in cellular organization, including pro tumorigenic genes, as well as cytopathic pathways, immune response mechanisms, response to viral infection, and protein processing (Fig. 5). In addition, 33 proviral genes were identified as having lost their function including endogenous retrovirus group members ERVV-2 and ERVMER34–1, Bcl2/-adenovirus receptors, influenza virus NS1A binding protein (IVNS1ABP), and angiotensin I converting enzyme 2 (ACE2) involved in SARS-CoV-2 cell entry mechanism^8^ (Table 2).

**Table 1:** Variant type-based distribution of genes with a predicted loss of function.

Variant caller	Variant type	Number of genes with predicted loss of function
Manta	Feature ablation	551
	Exon loss	38
	Frameshift	27
	Transcript ablation	12
	Gene fusion	3
SNVSniffer	Frameshift	8894
	Stop gained	592
	Splice donor	390
	Start lost	130

**Fig. 6:**
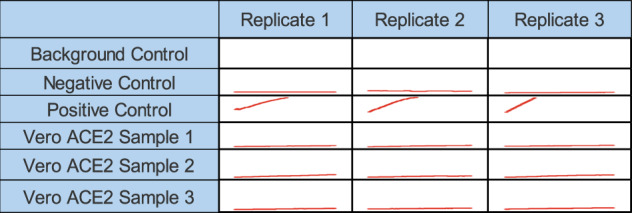
ACE2 enzymatic activity assessment across Vero ACE2 samples. Vero ACE2 activity assay Reading Matrix (Fluorometric).

**Table 2:** Proviral genes which lost their function due to structural variants.

NCBI ID	Gene name	LOF related variant type
103235187	endogenous retrovirus group V member 2(ERVV-2)	Deletion: Frameshift variant
103231639	angiotensin I convening enzyme 2(ACE2)	Insertion: Splice acceptor & intron variant (multiple stop codons) Deletion: Frameshift variant
103227905	endogenous Bomavirus-like nucleoprotein 2(EBLN2)	Insertion: Frameshift variant (multiple stop codons)
103229363	feline leukemia virus subgroup C cellular receptor family member 2(FLVCR2)	Insertion: Frameshift variant
103225011	human immunodeficiency virus type I enhancer-binding protein 3(HIVEP3)	Deletion: Frameshift variant
103230431	influenza virus NSIA binding protein(IVNSIABP)	Insertion: Frameshift variant
103240251	murine retrovirus integration site 1 homolog(MRVI1)	Deletion: Feature ablation (multiple stop codons)
103235692	endogenous retrovirus group MER34 member HERVMER34–1)	Deletion: Frameshift variant (multiple stop codons)
103237620	solute carrier family 52 member 2(SLC52A2)	Insertion: Frameshift variant
103215978	solute carrier family 52 member 3(SLC52A3)	Deletion: Frameshift variant
103227211	zinc finger and SCAN domain containing 10(ZSCANIO)	Insertion: Frameshift variant
103222367	zinc finger and SCAN domain containing 12(ZSCANI2)	Deletion: Frameshift variant
103221929	zinc finger and SCAN domain containing 16(ZSCANI6)	Deletion: Frameshift variant
103246758	zinc finger and SCAN domain containing 21(ZSCAN21)	Insertion: Frameshift variant
103221914	zinc finger and SCAN domain containing 23(ZSCAN23)	Insertion: Frameshift variant
103245744	zinc finger and SCAN domain containing 29(ZSCAN29)	Insertion: Frameshift variant
103227463	zinc finger and SCAN domain containing 32(ZSCAN32)	Insertion: Frameshift variant
103235415	zinc finger and SCAN domain containing 4(ZSCAN4)	Deletion: Frameshift variant
103227450	zinc finger protein 174(ZNF174)	Insertion: Frameshift variant
103227495	zinc finger protein 197(ZNF197)	Insertion: Frameshift variant (multiple stop codons)
103248713	zinc finger protein 202(ZNF202)	Insertion: Frameshift variant
103241144	zinc fmger protein 215(ZNF215)	Deletion: Frameshift variant (no start codon)
103242227	zinc finger protein 232(ZNF232)	Deletion: Frameshift variant
103222539	zinc finger protein 24(ZNF24)	Insertion: Frameshift variant
103227313	zinc fmger protein 263(ZNF263)	Deletion: Frameshift variant
103222546	zinc finger protein 397(ZNF397)	Deletion: Frameshift variant
103235347	zinc finger protein 444(ZNF444)	Deletion: Frameshift variant
103227490	zinc finger protein 445(ZNF445)	Deletion: Frameshift variant
103235451	zinc fmger protein 446(ZNF446)	SNP: Splice acceptor & intron variant
103221931	zinc finger with KRAB and SCAN domains 8(ZKSCANS)	Deletion: Frameshift variant
103233722	ATCAY, caytaxin(ATCAY)	Insertion: Frameshift variant
103247216	BCL2 interacting protein-like(BNIPL)	Insertion: Frameshift variant
103233646	adapter related protein complex 3 delta 1 subunit(AP3D1)	

*LOF* loss of function.

### Identification of viral sequences

Following a BLASTN^18^ search on the custom-made viral sequences database, several viral genomic sequences were identified for both Vero genome pseudohaplotypes and the African Green Monkey genome (Table 3) with an E-value cutoff of 10^–50^ to account for only highly similar sequences. These sequences include as expected retroviral sequences such as the simian retroviral^19^ and human endogenous retroviruses. In addition, complete viral genomes of the Adeno-associated virus serotypes 3, 4, 7, 8, sarcomas, blastomas, and leukemia viruses were identified, among others.

**Table 3:** Viral genomic sequences inserted in the Vero cell line genome.

RefSeq release number	Viral sequence	E-value
NC_002665.1	Bovine herpesvirus 4 long unique region, complete sequence	0.0
NC_009889.1	RD114 retrovirus, complete genome	0.0
NC_022518.1	Human endogenous retrovirus K113 complete genome	0.0
NC_031326.1	Simian retrovirus 8 strain SRV8/SUZ/2012, complete genome	0.0
NC_014474.1	Simian retrovirus 4 strain SRV4/TEX/2009/V1, complete genome	0.0
NC_022517.1	Baboon endogenous virus strain M7 proviral DNA, complete genome	0.0
NC_001550.1	Mason-Pfizer monkey virus, complete genome	0.0
NC_001829.1	Adeno-associated virus - 4, complete genome	0.0
NC_001729.1	Adeno-associated virus - 3, complete genome	0.0
NC_006260.1	Adeno-associated virus - 7, complete genome	1E-175
NC_006261.1	Adeno-associated virus - 8, complete genome	6E-164
NC_001499.1	Abelson murine leukemia virus, complete genome	5E-163
NC_001350.1	Saimiriine herpesvirus 2 complete genome	4E-132
NC_032111.1	BeAn 58058 virus, complete genome	2E-114
NC_038922.1	Avian sarcoma virus CT10 genomic sequence	8.00E-97
NC_038858.1	FBR murine osteosarcoma, complete proviral sequence	2.00E-83
NC_001506.1	Murine osteosarcoma virus, complete genome	2.00E-83
NC_009424.5	Woolly monkey sarcoma virus	2.00E-81
NC_041925.1	Proteus phage VB_PmiS-Isfahan, complete genome	5.00E-79
NC_003678.1	Pestivirus giraffe-1 H138 complete genome	1.00E-66
NC_008094.1	Y73 sarcoma virus, complete genome	2.00E-66
NC_001461.1	Bovine viral diarrhea virus 1, complete genome	5.00E-65
NC_043404.1	Avian myeloblastosis virus RNA-dependent DNA polymerase gene, partial cds; transforming protein gene, complete cds; and long terminal repeat, complete sequence.	2.00E-63
NC_043382.1	Snyder-Theilen feline sarcoma virus genomic sequence	1.00E-59
NC_038923.1	Hardy-Zuckermann 4 feline sarcoma virus (H24-FeSV) kit oncogene	3.00E-57
NC_038668.1	Harvey murine sarcoma virus p21 v-has protein gene	5.00E-54
NC_001885.3	Gibbon ape leukemia virus gag, pol, and env genes, complete cds	1.00E-50
NC_007815.2	PreXMRV-1 provirus, complete genome	5.00E-50

### ACE2 preliminary analyses

A comparison of Vero ACE2(vACE2) and human ACE2(hACE2) protein sequences showed 43 residues mutations (Tables 4), 94.71% identity, and respectively, a molecular weight of 92,427 and 92,463 Da for vACE2 and hACE2. As shown in Table 4, the mutations were highlighted on the protein 3D structure. A preliminary experiment to assess the loss of function prediction for ACE2 in Vero cells at the final gene product level was conducted. Indeed, ACE2 activity was assessed via the activity assay and vACE2 cells showed no activity for three different cell culture samples in triplicates (Fig. 6).

**Table 4:** Residues mutations between vACE2(alteration) and hACE2(original) and mutations positions on vACE2 3D structure(red).

Position	Original	Alteration	Position on vCAE2 3D structure
3	S	G	
67	D	E	
87	E	A	
136	D	N	
145	E	D	
153	A	E	
154	N	K	
167	S	G	
197	E	K	
218	S	N	
220	G	D	
228	H	R	
259	I	T	
299	D	N	
303	D	N	
342	A	V	
359	L	I	
555	F	L	
559	R	K	
630	D	A	
631	R	N	
634	E	K	
657	K	E	
658	V	N	
660	N	H	
662	M	T	
674	N	D	
684	F	Y	
702	K	E	
706	M	F	
716	R	Q	
729	P	S	
732	G	V	
735	N	Y	
737	P	S	
739	V	I	
740	S	T	
741	I	T	
751	G	A	
755	V	A	
759	I	V	
773	K	Q	
777	G	E	

## Discussion

In this work, we present a de novo assembly and annotation of two pseudohaplotypes for the Vero cell line, providing a genomic tool for a better understanding of the Vero cell line and its interactions with viruses but also for the design of more efficient cell engineering strategies such as CRISPR^20^ screenings and gene editing(by proposing an annotated Vero genome that can be used as a baseline for screening approaches such as more rigorous gene expression profiling, pathway enrichment analysis and even the generation of a CRISPR screening library for Vero cells). Given the identification of Vero cells as an aneuploid cell line, it is necessary to fully characterize the heterogeneity of the Vero cell population using tools such as single-cell analysis. Indeed, bulk genome analysis of cell populations tend to conceal differences between changes in expression from changes in the cellular composition of the population. Thus, single-cell analysis has emerged as a tool to have a detailed understanding of genomic, epigenomic, and transcriptional variations at the single-cell level in order to detect cellular heterogeneity and more precisely identify major subpopulations emerging from that heterogeneity. Due to the lack of a fully annotated reference genome to assist in single-cell analysis, we took a first preliminary step proposing this haplotype-resolved draft assembly genome giving a first glance into the heterogeneity of this continuous cell line by comparing the two haplotypes. Comparing this assembly with the African Green Monkey^10^ genome helped unravel genomic events explaining Vero cell characteristics as a cell culture platform. It is necessary to note that despite the stringent statistical parameters used for variant calling, errors due to sequencing technologies can still affect variant calls, thus loss of function predictions. Therefore, it is necessary for end users of this draft assembly to validate variants related to their genes of interest before downstream applications.

Notably, the interchromosomal translocations previously discovered via karyotyping^10^, were identified alongside other large-scale and small-scale structural variants through variant calling (Fig. 4 and Supplementary Data 4) and can be used to fully characterize the interchromosomal events in Vero cells. Furthermore, the effects of those genomic rearrangements on gene products functionality further explained the continuous nature of the cell line and its relative high susceptibility to infection^8^. Indeed, several genes involved in the cytopathic pathway, cell regulations, immune response, and pro tumorigenic genes lost their function due to frameshift variants, features ablation, splice acceptor variant, and intron variants, among others.

In light of the extensive rearrangements observed both in our analysis and in the 2014 Landscape of Vero cells paper^10^, we don’t think that the African Green Monkey genome should be used as a reference to search for misassemblies and correct a genome assembly of the Vero cells because, if the African Green Monkey is used as a reference for that purpose, the extensive rearrangements will appear in the misassembly report and correcting those to comply with the reference, as per traditional reference-based genome assemblies, will not generate an assembly that accurately describes the Vero cells genome. As expected, given the significant amount of rearrangements, when running QUAST^21^ with the African Green Monkey as a reference, NGA50 is 1.2 Mb, which could be due to either significant genomic rearrangements or chimeric contigs present in either the African Green Monkey assembly or our Vero assembly. But, alignment of short reads to our assembly detected no chimeric read and given the extensive rearrangements observed in the 2014 Landscape paper^10^ and in our analysis (which are detailed in the Supplementary Data and visually shown in Fig. 4) are the main cause of such NGA50 value. Nonetheless, the African Green Monkey assembly is a valuable tool to identify rearrangements in the Vero cell genome compared to the African Green Monkey genome from which it is derived in order to shed some light on the peculiar characteristics of this cell line that makes it a valuable candidate for virus production and vaccine manufacturing.

In addition to the Simian retroviral sequence insertions previously identified^19^, the analysis of this Vero cell genome further showed the insertion of several complete viral genomes including the human endogenous retroviral sequence, proviral sequences, sarcoma viruses, and adeno-associated viruses which might provide insights on developing efficient downstream processing steps and quality control tools for manufacturing biologics. Furthermore, the availability of an annotated Vero cell line genome provides new possibilities for viral sequence clearance through gene editing. Also, the proviral genes identified as having lost their function are involved in key stages of retroviruses, leukemia viruses, influenza virus, and adenoviruses reproduction cycle despite Vero cells wide use as a susceptible to highly susceptible cell line. Given the success of the Vero cell line as a virus production platform, the predicted loss of function of proviral genes might appear as counterintuitive but these proviral genes being specific to their associated type of virus, on one hand, the loss of function might affect that specific virus production rate but other virus production rates might not be affected. For example, in the case of influenza virus strains, the Vero cell line was one of the first cell lines considered as a vaccine production platform for influenza vaccines but its relatively low viral particles production yield limited the use of the Vero cell line as a manufacturing platform. Our analysis of the Vero genome demonstrated that Influenza virus NS1A binding protein (IVNS1ABP) also lost its function, hindering the production of M2 protein and the overall Influenza virus production yield. Thus, with an annotated genome now available, new strategies can be designed to reengineer high-yield Vero cell line for influenza vaccine production. On the other hand, the predicted loss of function might hint towards an alternative cell–virus mode of interaction used by Vero cells to bypass this predicted loss of function and still produce the virus at high rates. This might be the case for ACE2 where despite the ACE2 gene (receptor for SARS-CoV and SARS-CoV-2 host cell entry^8^) predicted loss of function, the Vero cell line has a high susceptibility to SARS-CoV and SARS-CoV-2 infection and is consequently used for the production of inactivated and attenuated COVID-19 vaccines. It is important to note that the loss of function for ACE2 in Vero cells remains a prediction rather than a fact. While the ACE2 protein is predicted to at least lose its catalytic function in Vero cells which is corroborated by preliminary experimental results(Table 4, Fig. 6), further experiments are necessary to determine whether or not the receptor-binding function of the ACE2 protein is maintained in Vero cells, via, for instance, detailed mass-spectroscopy analyses of viral binding sites, which are beyond the scope of this paper. Overall, these proviral genes predicted loss of functions need to be investigated on a case-by-case viral infection in order to fully comprehend the Vero cell line as a viral production platform. Vero cell’s ability to be infected at high multiplicity of infection (MOI), without instantly triggering the cytopathic pathway, was first explained with the loss of type I interferon^10^, which was confirmed with the identification of genes involved in the pathway, from interferons and caspases loss to chemokines hindrances, losing their functions. In addition, a BLAST^16^ search against viral databases revealed the insertion of the BeAn 58058 virus complete genome which contains cytokine response-modifying protein B, surface antigen S, chemokine-binding protein, interferon antagonist K1L, and serine proteinase inhibitor 1, among other cell death inhibitors, thus strengthening the cytopathic pathway inhibition. Gene profiling of infected Vero cells might provide additional insights on the balance between the effect of the host cell antiviral genes loss of function and the insertion of virus proviral gene sequences into the host cell genome.

Furthermore, the adeno-associated complete viral sequences insertion into the Vero genome might provide new alternatives for the study of Vero cell-based adeno-associated virus and eventually design of alternative production platforms for AAV serotypes.

In addition, gene-editing tools have been used to create Vero suspension cell lines, which are considered to facilitate bioprocess development efforts. Nevertheless, singular genome modifications did not seem to lead to successful and sustainable results^1^. Using the annotated genome presented here, further studies can be conducted to investigate suspension adaptation efforts, for example, large-scale screens could identify if a combination of modifications is needed. Additionally, already adapted suspension Vero cell lines still exhibit low cell growth rates with doubling times of more than 40 h and the frequent formation of aggregates^22^. Genome-wide screens or comparative transcriptomic studies could further investigate factors that would lead to improved Vero suspension systems.

In the long run, the findings of this study and previous ones^6,19^ are sought to pave the way for the widespread application of genome analysis, screening, and editing tools for the Vero cell line. Taking into account the use of Vero cells in vaccine manufacturing processes and in particular the acceptance of this cell line by regulatory authorities, successful applications of genome editing can significantly improve virus production and ultimately lower the cost of vaccine manufacturing.

## Methods

### Cell lines and culture media

The Vero WHO cell line studied in this work was at passage 138(Neovacs). This cell line was itself derived from a vial of Vero ATCC CCL-81 which was sent to WHO at passage 124 for analysis and establishment of the Vero WHO master cell bank approved for vaccine production. The cells were grown in the static culture at 37 °C and 5% CO_2_ in a humidified incubator (Infors HT, Switzerland). Cells were passaged twice weekly using TrypLE Express (Thermo Fisher Scientific) as a dissociation reagent. A serum-free adapted sub-cell line grown in OptiPRO medium (Thermo Fisher Scientific) supplemented with 4 mM GlutaMAX (Thermo Fisher Scientific) was cryopreserved at a passage number of 151 in OptiPRO medium supplemented with 4 mM GlutaMAX and 10% DMSO (Sigma, USA).

For genome analysis, Vero WHO cells at passage 153 were washed in PBS (Wisent, Canada), harvested using TrypLE Express, and centrifuged at 300×*g* for 5 min. Cell pellets of around 6 million cells were quickly frozen in a mixture of dry ice/ethanol and stored at −80 °C until further analysis.

### De novo genome assembly and annotation

The 10x Genomics linked read libraries were sequenced on three HiSeqX lanes with paired-end 151 reads. The reads were first processed with 10x Genomics Long Ranger^23^ basic to flag all the reads with a valid molecule barcode. These processed reads were then used to filter the original demultiplexed reads keeping only the reads that carry valid barcodes. The assembly was then run using 10x Genomics Supernova^24^ run with the following options: “—bcfrac=1—maxreads=1490 M—localcores=16—localmem=327”. Following the assembly step fasta files with the assembly sequence were generated using Supernova mkoutput with these options: “—style = pseudohap2—minsize=250—headers = full” to generate a principal and an alternate pseudohaplotype. 10x Genomics assembler, Supernova, initially outputs the assembly in the form of a graph where edges are assembled sequences. These sequences are linked together at the ends by overlaps of K-1 bases (K = 48). To transform the assembly graph into fasta format the graph can be traversed concatenating the sequences along each visited edge. When using the pseudohap Supernova option to extract the fasta version of the assembly, the graph is traversed once based on the highest coverage edges. With the pseudohap2 option the traversal is performed by choosing the second-highest coverage edges. This results in two very similar assembly files that differ only where large variations are present.

With the barcode information generated during 10X Linked Read sequencing protocol, ARCS^25^, pipelined with LINKS^26^ and Tigmint^27^, was used to pair the Supernova draft assembly sequences by processing input alignments for sets of read pairs from the same barcode that aligned to different sequences and formed a link between sequence contigs. The iteration parameters m = 50–20000/e = 90000 and m = 50–10000/e = 30000 were applied for respectively the principal pseudohaplotype and the alternate pseudohaplotype. Following this scaffolding protocol, Nanopore long reads were used for gap filling via RAILS/Cobbler^28^ (iteration parameters: i = 0.7, 0.65/d = 250–50/l = 1 for the principal pseudohaplotype; i = 0.7,0.65/d = 250–10/l = 1 for the alternate pseudohaplotype) then the scaffolds were polished with ntEdit^28^ (k = 64,50,40). The assembly quality control metrics were calculated using QUAST^29^ and a preliminary gene prediction was done via AUGUSTUS^30^.

The principal pseudohaplotype annotation was performed using NCBI’s in-house Eukaryotic Annotation Pipeline^31^.

The QV value was calculated using Merqury^14^ to first assess the optimal k-mer value based on the genome size, followed by Meryl^15^ short read database build and QV evaluation.

### Genomic rearrangements detection

Previously generated interleaved reads were mapped to the African Green Monkey annotated genome^10^ using the BWA-MEM algorithm^32^ and the resulting BAM file was generated via SAMTOOLS^33^ view. Deeptools^34^ was used to plot the genome coverage. Large-scale indels, duplication tandem, and interchromosomal translocations were called using Manta^17^ with default parameters. SNVSniffer^16^ was used to call the remaining small-scale indels and SNPs (exec_mode parameter = 2 for most accurate variant calling where following the variant call, all reads are realigned to calculate per-base alignment quality (BAQ) scores before inputting those alignments to the calling engine^16^). Besides the description of Vero cells as an aneuploid cell line and its major karyotyping presented in the Landscape of Vero cells^6^, given the lack of additional information on Vero cells heterogeneity, we decided to use variant caller that are designed for both somatic and germline variant calls (SNVSniffer^16^ and Manta^17^) and apply their suggested default parameters for variant calls. In addition, both pipelines score variant candidates relative to the reference to identify and filter out (if needed) variants due to the Vero cell genome heterogeneity.

The effect of those called variants were predicted using Galaxy’s SNPEff^35^ to extract all genes that lost their functions. Those resulting genes were functionally annotated via DAVID^36^, filtered, and clustered in biological groups. Large-scale structural variants called Manta were plotted via Circos^37^. Variant calls statistics were calculated for both SNVSniffer and Manta using bcftools stats^38^.

### Viral genomic sequences detection

To identify and characterize the viral genomic insertions in the Vero genome, all viral sequence releases from RefSeq were used to create a blastn database and a BLAST^18^ search was run for both Vero genome pseudohaplotypes. To ensure that no false positives were included in the results, the African Green Monkey genome^10^ was also run against the created viral database and all resulted in viral sequences were identified in both the Vero genome and the African Green Monkey genome.

### ACE2 preliminary analyses

Vero ACE2 protein sequence was obtained from NCBI annotation of our assembly (NCBI *Chlorocebus sabaeus* (Vero cell) Annotation Release 102 (AR 102)) and a BLAST^18^ search was run on the NCBI portal to identify residues mutations. The 3D structure of vACE2 was modeled using Phyre2 server^39^, mutations were marked using EzMol^40^.

For ACE2 activity assessment, three separate cultures of Vero cells were prepared in the same conditions and used with Abcam Angiotensin II Converting Enzyme (ACE2) Activity Assay Kit (Fluorometric) (ab273297) as per the provided protocol all conditions were analysed in triplicates. Fluorometric reading was performed for an hour at 1 min intervals.

### Reporting summary

Further information on research design is available in the Nature Research Reporting Summary linked to this article.

## Supplementary information

Supplementary Information

Supplementary Data 1

Supplementary Data 2

Supplementary Data 3

Supplementary Data 4

Reporting Summary

## Data Availability

The assembly and annotation data have been deposited in NCBI under the accession numbers JACDXN000000000 and JACDXO000000000, for the principal and alternate pseudohaplotypes respectively. The NCBI *Chlorocebus sabaeus* (Vero cell) Annotation Release 102 can be directly accessed through the following page: https://www.ncbi.nlm.nih.gov/genome/annotation_euk/Chlorocebus_sabaeus/102/. The full comparison table between the Vero annotation 102 and the African Green Monkey annotation 100 can be downloaded through the following link: https://ftp.ncbi.nlm.nih.gov/genomes/all/annotation_releases/60711/102/GCF_015252025.1_Vero_WHO_p1.0/Annotation_comparison/GCF_015252025.1_Vero_WHO_p1.0_compare_prev.txt.gz.

## Code availability

This study did not make use of custom code.
