# Supplementary material for: Haplotype-resolved de novo assembly of the Vero cell line genome
Source: NPJ Vaccines. 2021 Aug 20;6:106. doi: 10.1038/s41541-021-00358-9 (PMC8379168; doi:10.1038/s41541-021-00358-9)
Supplement: Supplementary file 1 — Supplementary Information [file 41541_2021_358_MOESM1_ESM.pdf]

**Supplementary Information Guide**

**Supplementary File 1:** Preliminary scaffolding optimization and additional validation evidences

**Supplementary Data 1:** List of functionally annotated genes predicted as having lost their function

**Supplementary Data 2:** SNVSniffer loss of function variants full annotation and loss of function prediction

**Supplementary Data 3:** Manta loss of function variants full annotation and loss of function prediction

**Supplementary Data 4:** Large-scale variants annotations including interchromosomal changes

# Supplementary File 1

# Content

Preliminary optimization of scaffolding metrics……………………………………………………………………………………….p1

Comparison between breaks in the Vero genome assembly and the African Green Monkey assembly….p8

Viral insertions validation: Analysis of reads spanning the junctions on both ends of the insertions………p9

Validation of ACE2 partial loss of function from evidences other than the assembly……………………………..p11

Additional validations using RNA-seq data……………………………………………………………………………………………..p13

# Preliminary optimization of scaffolding metrics

## Step 1: First draft assembly

The 1.49 billion reads generated through Illumina sequencing were first filtered to discard reads without barcodes, then processed for first draft assembly using Supernova assembler at a 75X coverage. Two output haplotypes were generated (named Vero1 and Vero2) and processed for quality assessment using QUAST (Supplementary Figures 1-4) before proceeding to scaffolding. N50 (Supplementary Table 1), which is defined as a median length of a set of contigs, is the most common metrics used for de novo assembly quality assessment.

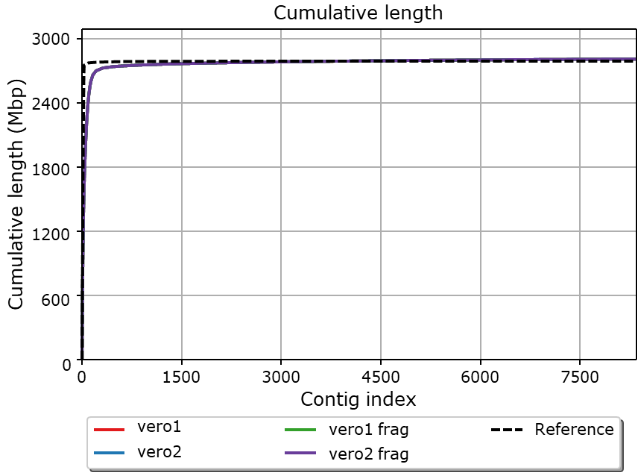


*Supplementary Figure 1: Cumulative length plot ( growth of contig lengths)*

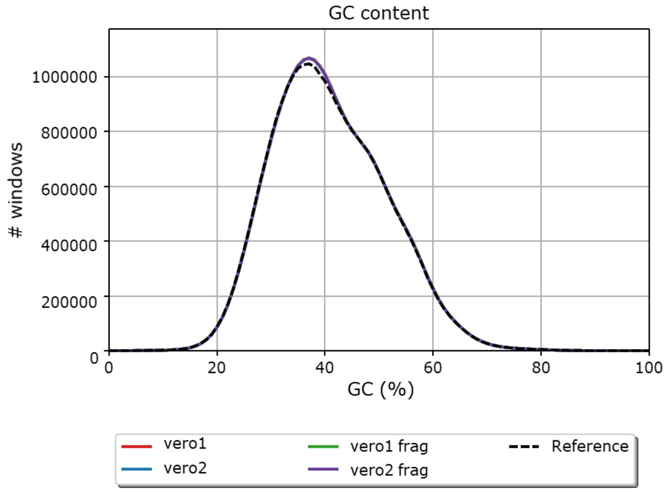


*Supplementary Figure 2: GC content plot shows the distribution of GC content in the contigs*

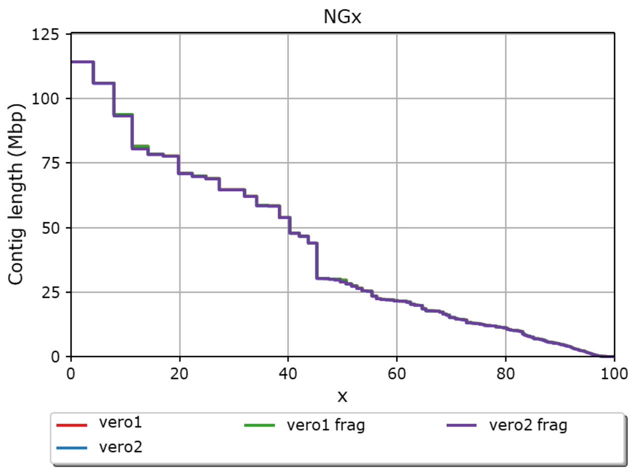


*Supplementary Figure 3: NGx plot (NGx values as x varies from 0 to 100 %)*

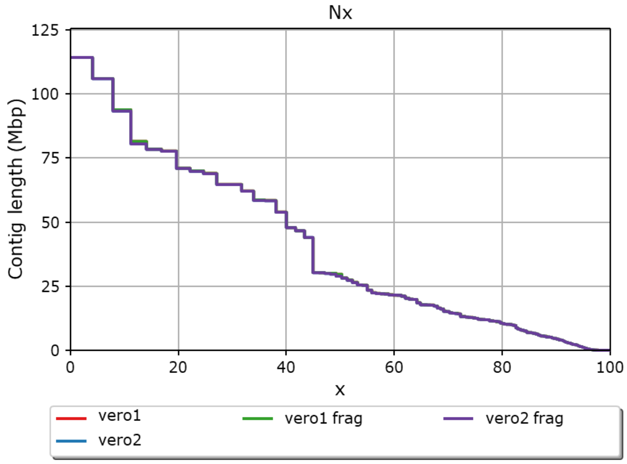


*Supplementary Figure 4: Nx plot (Nx values as x varies from 0 to 100 %)*

Worst Median Best ☑ Show heatmap

| Statistics without reference | test_data_vero1 | test_data_vero2 |
|---|---|---|
| # contigs | 8351 | 8350 |
| # contigs (>= 0 bp) | 56 471 | 56 471 |
| # contigs (>= 1000 bp) | 19 713 | 19 713 |
| # contigs (>= 5000 bp) | 5195 | 5191 |
| # contigs (>= 10000 bp) | 2507 | 2499 |
| # contigs (>= 25000 bp) | 955 | 950 |
| # contigs (>= 50000 bp) | 494 | 494 |
| Largest contig | 114 161 801 | 114 260 507 |
| Total length | 2 810 851 524 | 2 808 019 323 |
| Total length (>= 0 bp) | 2 847 827 728 | 2 844 998 449 |
| Total length (>= 1000 bp) | 2 829 937 088 | 2 827 107 809 |
| Total length (>= 5000 bp) | 2 798 796 555 | 2 795 956 127 |
| Total length (>= 10000 bp) | 2 779 992 876 | 2 777 118 082 |
| Total length (>= 25000 bp) | 2 756 656 200 | 2 753 828 193 |
| Total length (>= 50000 bp) | 2 740 958 612 | 2 738 279 984 |
| N50 | 29 636 809 | 28 939 313 |
| N75 | 12 633 392 | 12 631 281 |
| L50 | 23 | 23 |
| L75 | 60 | 60 |
| GC (%) | 40.85 | 40.85 |
| **Mismatches** | | |
| # N's | 38 271 870 | 38 234 790 |
| # N's per 100 kbp | 1361.58 | 1361.63 |

*Supplementary Table 1: Summary report*

Step 2: Final draft assembly

Following quality assessment of the first draft contigs assembly, scaffolding is performed to improve the assembly metrics and completeness. By using the barcode information generated during 10X Linked Read sequencing protocol, a linked read kmers-based mapping approach was employed for draft assembly sequences ordering and orientation and gap sizes estimations.

ARCS, pipelined with LINKS and tigmint via the Makefile arcs-make, is used to pair the Supernova draft assembly sequences by processing input alignments for sets of read pairs from the same barcode that aligned to different sequences and formed a link between sequence contigs. Barcode sequencing errors are accounted for by selecting only barcodes within a specified multiplicity range (parameter m) and a specified maximum window length at the end sequences, where Chromium reads align (parameter e). Once the optimal candidates value for m and e for scaffolding were chosen, the actual pairing of those contigs was done using LINKS.

After running several scaffolding experiments to determine the optimal parameters (m and e) (Supplementary Figures 5-6, Supplementary Table 2), the optimal parameters combination was set at m=50-20000 and e=90000.

In order to further improve the scaffolding an iteration strategy was designed using the output of the previous scaffolding experiment and applying to it the selected optimal parameters as illustrated in Supplementary Figure. 7-9 while evaluating the output at each step by running QUAST and BUSCO search for sanity check. The BUSCO completeness was constant during the iterations at 87.5% for pseudohaplotype 1 and 88.4% for pseudohaplotype 2.

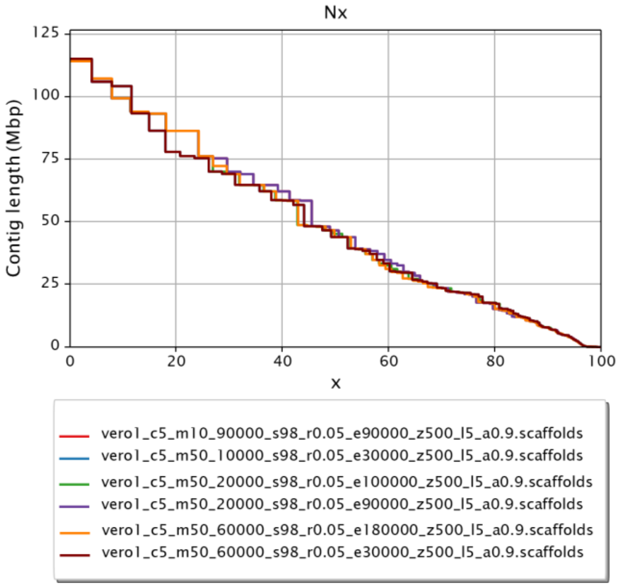


*Supplementary Figure 5: Nx comparison plot for optimal parameter selection (Nx values as x varies from 0 to 100 %)*

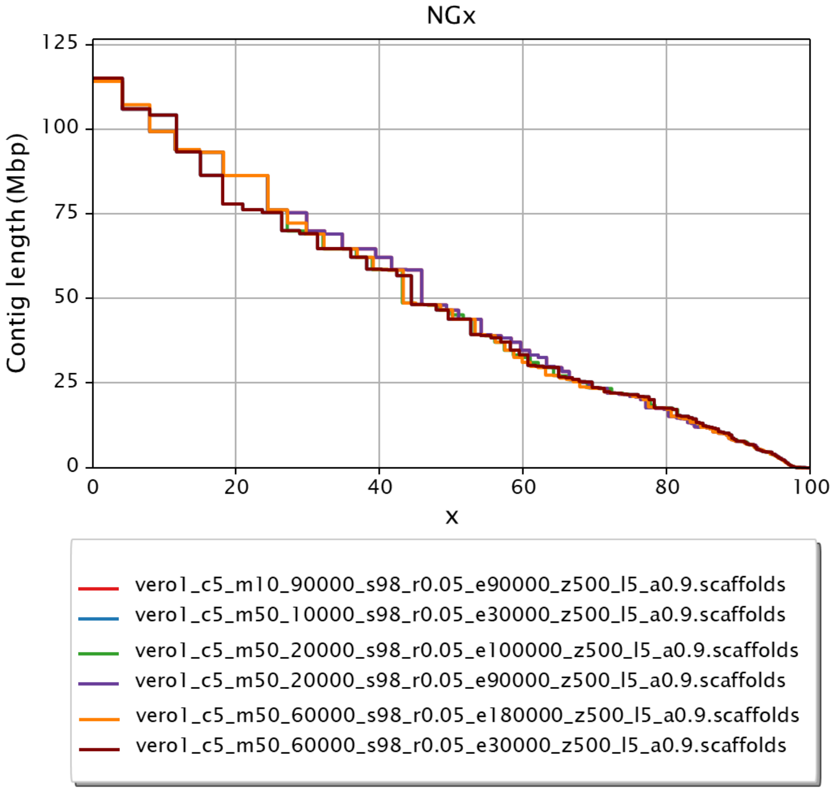


*Supplementary Figure 6: NGx comparison plot for optimal parameter selection (NGx values as x varies from 0 to 100 %)*

| | vero1_c5_m10_90000_s98_r0.05_e90000_z500_l5_a0.9.scaffolds | vero1_c5_m50_10000_s98_r0.05_e30000_z500_l5_a0.9.scaffolds | vero1_c5_m50_20000_s98_r0.05_e100000_z500_l5_a0.9.scaffolds | vero1_c5_m50_20000_s98_r0.05_e90000_z500_l5_a0.9.scaffolds | vero1_c5_m50_60000_s98_r0.05_e180000_z500_l5_a0.9.scaffolds | vero1_c5_m50_60000_s98_r0.05_e30000_z500_l5_a0.9.scaffolds |
|---|---|---|---|---|---|---|
| # contigs (>= 0 bp) | 56047 | 56039 | 56046 | 56047 | 56076 | 56039 |
| # contigs (>= 1000 bp) | 19293 | 19285 | 19292 | 19293 | 19322 | 19285 |
| # contigs (>= 5000 bp) | 4808 | 4803 | 4806 | 4808 | 4836 | 4803 |
| # contigs (>= 10000 bp) | 2135 | 2132 | 2134 | 2135 | 2162 | 2132 |
| # contigs (>= 25000 bp) | 666 | 674 | 665 | 666 | 677 | 674 |
| # contigs (>= 50000 bp) | 316 | 317 | 316 | 316 | 325 | 317 |
| Total length (>= 0 bp) | 2847870128 | 2847870928 | 2847870228 | 2847870128 | 2847867228 | 2847870928 |
| Total length (>= 1000 bp) | 2829982338 | 2829983138 | 2829982438 | 2829982338 | 2829979438 | 2829983138 |
| Total length (>= 5000 bp) | 2798926625 | 2798936494 | 2798922149 | 2798926625 | 2798918337 | 2798936494 |
| Total length (>= 10000 bp) | 2780250737 | 2780270514 | 2780256652 | 2780250737 | 2780236990 | 2780270514 |
| Total length (>= 25000 bp) | 2758385626 | 2758576828 | 2758387489 | 2758385626 | 2758083893 | 2758576828 |
| Total length (>= 50000 bp) | 2746637640 | 2746568621 | 2746667943 | 2746637640 | 2746277735 | 2746568621 |
| # contigs | 7952 | 7946 | 7951 | 7952 | 7982 | 7946 |
| Largest contig | 114181557 | 114998122 | 114181557 | 114181557 | 114182697 | 114998122 |
| Total length | 2810939651 | 2810944662 | 2810939751 | 2810939651 | 2810938591 | 2810944662 |
| GC (%) | 40.85 | 40.85 | 40.85 | 40.85 | 40.85 | 40.85 |
| N50 | 46620840 | 43937327 | 45233560 | 46620840 | 43932119 | 43937327 |
| N75 | 21130642 | 21625564 | 21130642 | 21130642 | 21130642 | 21625564 |
| L50 | 19 | 20 | 20 | 19 | 20 | 20 |
| L75 | 42 | 44 | 44 | 42 | 44 | 44 |
| # N's per 100 kbp | 1363.05 | 1363.08 | 1363.06 | 1363.05 | 1362.95 | 1363.08 |

*Supplementary Table 2: Comparison between sets of scaffolding parameters efficiency*

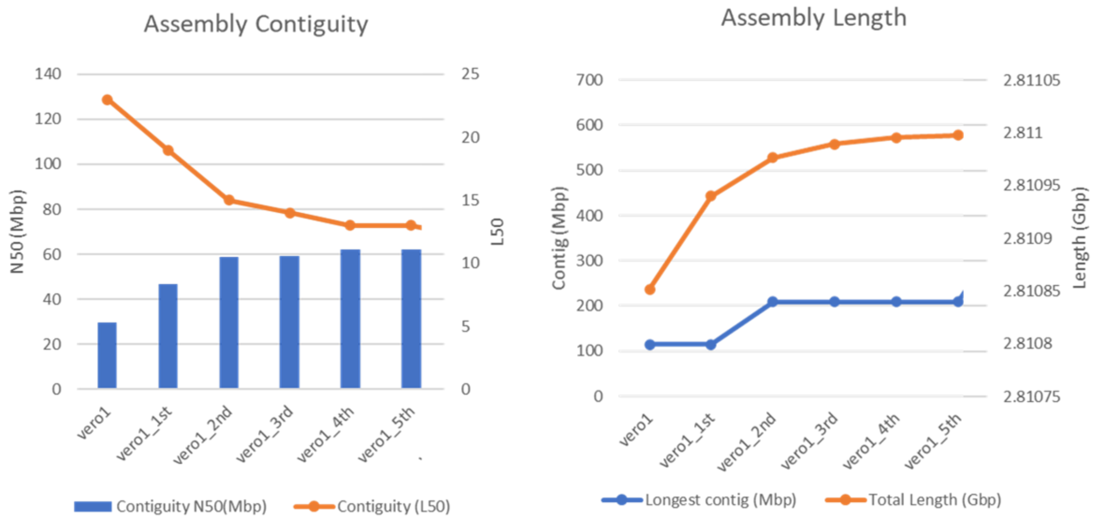


*Supplementary Figure 7: Sanity check of assembly quality (contiguity/length)*

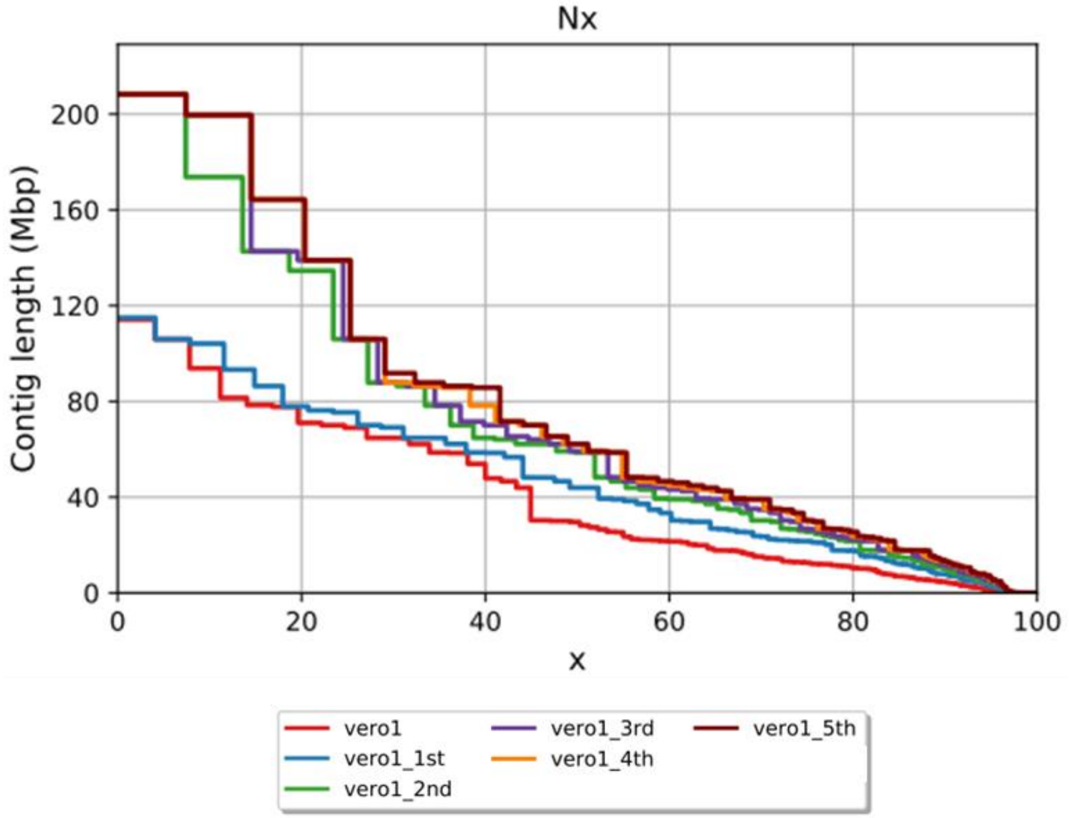


*Supplementary Figure 8: Nx comparison between iterations and first draft assembly plot (Nx values as x varies from 0 to 100 %)*

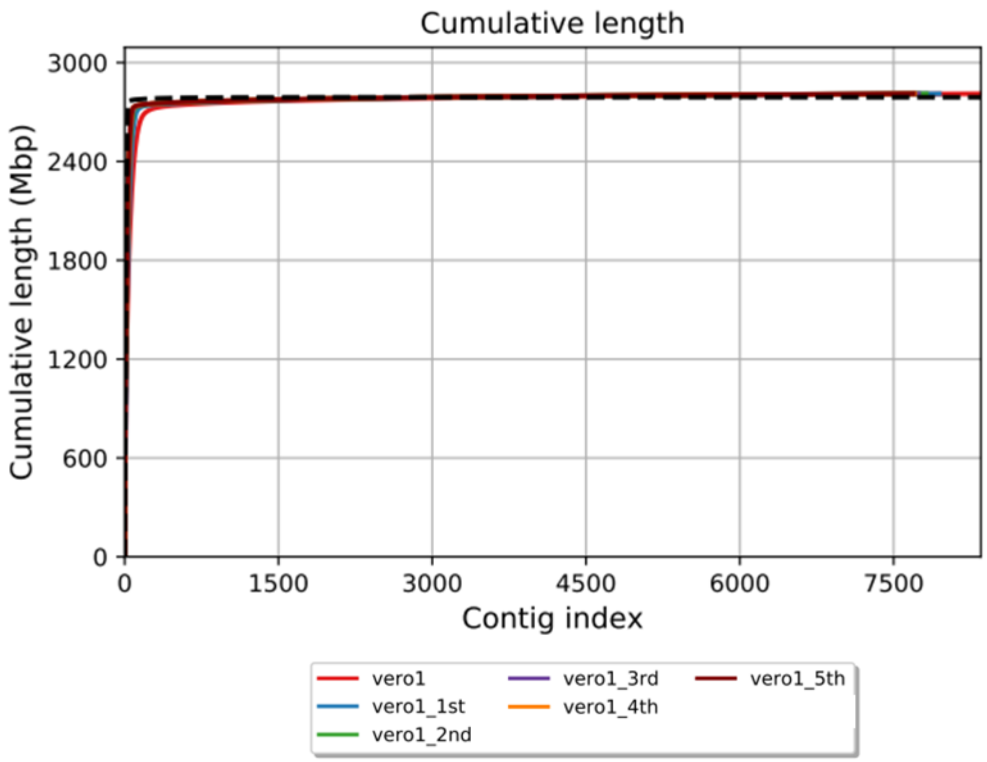


*Supplementary Figure 9: Cumulative length plot*

## Comparison between breaks in the Vero genome assembly and the African Green Monkey assembly

Alignment of large contigs of our Vero assembly to the African Green Monkey genome showed that none of the large contigs of both assemblies have full-length alignments to each other and share breaks as shown in the Supplementary Table below:

| African Green Monkey(AGM) Scaffold Length (bp) | Vero scaffold length (bp) | Alignment starting position on AGM scaffold | End alignment snapshot |
|---|---|---|---|
| 130588469 | 81790585 | 36270872 | 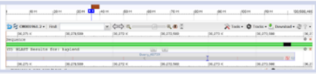 |
| 82825804 | 64349049 | 14176827 | 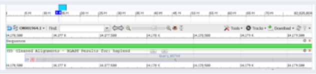 |
| 54310 | 211624478 | 35554 | 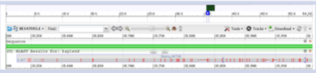 |
| 75399963 | 99694171 | 31382396 | 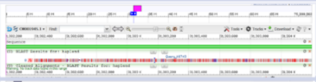 |
| 127223203 | 180281258 | 3066654 | 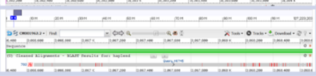 |
| 127223203 | 82484710 | 22387562 | 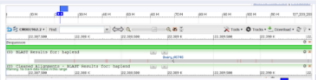 |
| 48547382 | 90413263 | 43538320 | 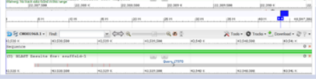 |
| 84932903 | 71207655 | 83565347 | 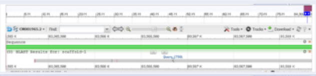 |
| 72318688 | 88423403 | 12028 | 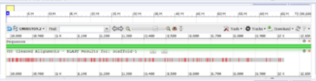 |
| 130038232 | 68151223 | 130031187 | 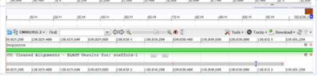 |

*Supplementary Table 3: Comparison between breaks in the Vero genome assembly and the African Green Monkey assembly*

## Viral insertions validation: Analysis of reads spanning the junctions on both ends of the insertions

For every viral insertion highlighted in our paper, our data included individual reads that span the junctions from viral to monkey sequence as shown in the following Supplementary Table:

| Viral genome insertions | Number of Vero scaffolds with buried viral genome | Number of reads spanning (log base 2) |
|---|---|---|
| **Bovine herpesvirus 4 long unique region, complete sequence** | 1 | 988 |
| **RD114 retrovirus, complete genome** | 85 | 199 |
| **Human endogenous retrovirus K113 complete genome** | 78 | 232 |
| **Simian retrovirus 8 strain SRV8/SUZ/2012, complete genome** | 59 | 84 |
| **Simian retrovirus 4 strain SRV4/TEX/2009/V1, complete genome** | 4 | 16 |
| **Baboon endogenous virus strain M7 proviral DNA, complete genome** | 92 | 2145 |
| **Mason-Pfizer monkey virus, complete genome** | 71 | 1274 |
| **Adeno-associated virus - 4, complete genome** | 1 | 4334 |
| **Adeno-associated virus - 3, complete genome** | 1 | 4334 |
| **Adeno-associated virus - 7, complete genome** | 1 | 4334 |
| **Adeno-associated virus - 8, complete genome** | 1 | 4334 |
| **Abelson murine leukemia virus, complete genome** | 1 | 2 |
| **Saimiriine herpesvirus 2 complete genome** | 1 | 25 |
| **Avian sarcoma virus CT10 genomic sequence** | 1 | 155 |
| **FBR murine osteosarcoma, complete proviral sequence** | 1 | 988 |
| **Murine osteosarcoma virus, complete genome** | 1 | 988 |
| **Woolly monkey sarcoma virus** | 1 | 57 |
| **Proteus phage VB_PmiS-Isfahan, complete genome** | 27 | 32 |
| **Pestivirus giraffe-1 H138 complete genome** | 1 | 3016 |
| **Y73 sarcoma virus, complete genome** | 1 | 128 |
| **Bovine viral diarrhea virus 1, complete genome** | 1 | 3016 |
| **Avian myeloblastosis virus RNA-dependent DNA polymerase gene, partial cds; transforming protein gene, complete cds; and long terminal repeat, complete sequence.** | 1 | 32 |
| **Snyder-Theilen feline sarcoma virus genomic sequence** | 1 | 155 |
| **Hardy-Zuckermann 4 feline sarcoma virus (H24-FeSV) kit oncogene** | 1 | 64 |
| **Harvey murine sarcoma virus p21 v-has protein gene** | 1 | 256 |
| **Gibbon ape leukemia virus gag, pol, and env genes, complete cds** | 1 | 105 |

*Supplementary Table 4: Analysis of reads spanning the junctions on both ends of the insertions*

In addition, these integrations are confirmed by the African Green monkey alignment. Moreover, NCBI independently identified the corresponding viral proteins using transcriptomic data from both our Vero RNAseq data and the vervet RNAseq data as shown in the following Supplementary Table:

| Source | Number of sequences retrieved from Entrez | Number (%) of sequences aligned by ProSplign | Number (%) of sequences passed to Gnomon | Average % identity | Average % coverage |
|---|---|---|---|---|---|
| Same-species GenBank | 182 | 147 (80.77%) | 147 (80.77%) | 79.85% | 94.03% |
| Same-species known RefSeq (NP_) | 31 | 28 (90.32%) | 28 (90.32%) | 82.58% | 91.97% |
| Primates GenBank | 21,649 | 14,932 (68.97%) | 14,932 (68.97%) | 80.50% | 89.77% |
| Primates known RefSeq (NP_) | 14,566 | 11,795 (80.98%) | 11,795 (80.98%) | 87.01% | 92.64% |
| Homo sapiens GenBank | 144,861 | 83,628 (57.73%) | 83,628 (57.73%) | 80.36% | 84.48% |
| Homo sapiens known RefSeq (NP_) | 60,894 | 45,057 (73.99%) | 45,057 (73.99%) | 87.45% | 91.32% |
| viral Other | 506 | 42 (8.30%) | 42 (8.30%) | 68.30% | 62.07% |

*Supplementary Table 5: NCBI alignments metrics*

## **Validation of ACE2 partial loss of function from evidences other than the assembly**

The ACE2 partial loss of function mutation (C to CTT) is subsequently validated in multiple ways:

1. Using **raw reads from DNA sequencing**: these reads were the data used to call the variants based on the mapping of the Vero genome DNA sequencing raw reads to the African Green Monkey genome assembly, thus our Vero genome assembly is not involved in this step.
2. Using **protein sequence** from NCBI annotation of our genome assembly: the 3D structure resulting from the protein sequence and the resulting mutations are not an inference from the assembly but generated by NCBI following their analysis of RNAseq evidence including the raw reads RNAseq data we submitted which is separated from our assembly thus providing an independent transcriptomics-based evidence.
3. Using ACE2 **transcripts** of both the Vero genome and the African Green Monkey genome: Indeed, concerning ACE2, NCBI has identified, through RNAseq data analysis and evidences from proteomics that the ACE2 transcript and protein sequences present changes between the African Green Monkey ACE2 and the Vero ACE2 which is an additional independent validation of our variant call for ACE2, which led to the assignment of a new specific transcript accession and protein accession numbers for Vero ACE2 to distinguish them from vervet ACE2 given their differences as shown below:

| previous gene range | previous gene strand | transcript category | current transcript accession | current protein accession |
|---|---|---|---|---|
| 14032230-14094162 | - | Change in UTR only | XM_007991113.2 | XP_007989304.2 |
| 14032230-14094162 | - | Variant | XM_037986357.1 | XP_037842285.1 |

*Supplementary Table 6: Variation in transcripts*

| current transcript range | previous transcript accession | previous protein accession | previous transcript range |
|---|---|---|---|
| 15866607-15915984 | XM_007991113.1 | XP_007989304.1 | 14032230-14094162 |
| 15866607-15912311 | XM_007991113.1 | XP_007989304.1 | 14032230-14094162 |

*Supplementary Table 7: Variation in transcripts*

4. Using **enzymatic activity assay**: we also previously validated the loss of function of the enzymatic activity and added the disclaimer that at this stage the loss of function of the receptor binding activity is not established and further analysis is needed which is out of the scope of this paper.

In addition, given the nature of the upstream variant and the downstream effect including loss of function predictions cannot be fully confirmed by PCR, as PCR might show the C to CTT variant but it is not enough to validate the loss of function, that work should be done at the proteomics level for a more solid proof as shown by our analysis and NCBI's transcriptomics and proteomics analysis. Nonetheless, the variant leading to ACE2 at least partial loss of function being a small scale insertion, it can be also validated by PCR but given the challenges of accurate primer design and false positives, in these cases it is recommended to analyse the PCR products via Sanger sequencing backed up with next-generation sequencing to compensate for the relatively

low efficiency of Sanger sequencing of PCR products and more precisely for SNPs and small indels call validation. In addition, the insertion is located in an intron which makes the use of mRNA challenging. Altogether, the validation of this variant via PCR might work but it will require significant optimization experiments before validations which is beyond the scope of this work and reasonable timelines.

5. Using **raw reads analysis**: As an alternative to PCR we further supported the variant call as suggested by the reviewer by using raw reads analysis: following mapping of RNAseq raw reads, we isolated the region containing the variant C to CTT and extracted all the reads that mapped to that region; all reads that mapped to that region contain the C to CTT variant.

## **Additional validations using RNA-seq data**

1. Ablated transcripts were identified for 1425 genes following RNAseq reads alignments and features count.
2. Following the use of RNAseq data for annotation and transcript characterization, only 1% of the genes in the Vero annotation are identical (i.e. Genes with perfect match in exon boundaries) to those of the African green monkey annotation, 46% of the genes had minor changes (i.e. Highly similar genes, with support scores of 0.66 or more (on a scale of 0 to 1) on both sides of the comparison. The support score is derived from a combination of matching exon boundaries and sequence overlap), 23% of the genes have major changes (i.e. Genes with support scores lower than 0.66 (on a scale of 0 to 1) on one or both sides of the comparison, and genes with changed locus, biotype or changed completeness, and split or moved genes), 30% of the genes are new (i.e. Novel genes or genes without a match in the African Green Monkey annotation). The link to the complete Supplementary Table comparing the Vero annotation with the African Green Monkey annotation was added in the data availability section. Notably, concerning ACE2, NCBI has identified, through RNAseq data analysis and evidence from proteomics that the ACE2 transcript and protein sequences present changes between the African Green Monkey ACE2 and the Vero ACE2 which is an additional independent validation of our variant call for ACE2 as shown below:

| previous gene range | previous gene strand | transcript category | current transcript accession | current protein accession |
|---|---|---|---|---|
| 14032230-14094162 | - | Change in UTR only | XM_007991113.2 | XP_007989304.2 |
| 14032230-14094162 | - | Variant | XM_037986357.1 | XP_037842285.1 |

*Supplementary Table 6: Variation in transcripts*

| current transcript range | previous transcript accession | previous protein accession | previous transcript range |
|---|---|---|---|
| 15866607-15915984 | XM_007991113.1 | XP_007989304.1 | 14032230-14094162 |
| 15866607-15912311 | XM_007991113.1 | XP_007989304.1 | 14032230-14094162 |

*Supplementary Table 7: Variation in transcripts*

3. Raw reads analysis also validated the C to CTT variant call resulting in ACE2 at least partial loss of
4. function in Vero cells with all reads mapped containing the variant.
5. In addition, the RNAseq data confirmed viral genome insertions by identifying viral transcripts and proteins: a total of 68 viral proteins (36 viral genes) were annotated.
