# Supplementary material for: Haplotype-resolved de novo assembly of the Vero cell line genome
Source: NPJ Vaccines. 2021 Aug 20;6:106. doi: 10.1038/s41541-021-00358-9 (PMC8379168; doi:10.1038/s41541-021-00358-9)
Supplement: Supplementary file 6 — Reporting Summary [file 41541_2021_358_MOESM6_ESM.pdf]

nature research

Corresponding author(s): Amine Kamen

Last updated by author(s): Feb 17, 2021

Reporting Summary

Nature Research wishes to improve the reproducibility of the work that we publish. This form provides structure for consistency and transparency in reporting. For further information on Nature Research policies, see Authors & Referees and the Editorial Policy Checklist.

Statistics

For all statistical analyses, confirm that the following items are present in the figure legend, table legend, main text, or Methods section.

n/a	Confirmed	
☒	☐	The exact sample size (*n*) for each experimental group/condition, given as a discrete number and unit of measurement
☒	☐	A statement on whether measurements were taken from distinct samples or whether the same sample was measured repeatedly
☒	☐	The statistical test(s) used AND whether they are one- or two-sided<br>*Only common tests should be described solely by name; describe more complex techniques in the Methods section.*
☒	☐	A description of all covariates tested
☒	☐	A description of any assumptions or corrections, such as tests of normality and adjustment for multiple comparisons
☒	☐	A full description of the statistical parameters including central tendency (e.g. means) or other basic estimates (e.g. regression coefficient) AND variation (e.g. standard deviation) or associated estimates of uncertainty (e.g. confidence intervals)
☒	☐	For null hypothesis testing, the test statistic (e.g. *F*, *t*, *r*) with confidence intervals, effect sizes, degrees of freedom and *P* value noted<br>*Give P values as exact values whenever suitable.*
☒	☐	For Bayesian analysis, information on the choice of priors and Markov chain Monte Carlo settings
☒	☐	For hierarchical and complex designs, identification of the appropriate level for tests and full reporting of outcomes
☒	☐	Estimates of effect sizes (e.g. Cohen's *d*, Pearson's *r*), indicating how they were calculated

Our web collection on statistics for biologists contains articles on many of the points above.

Software and code

Policy information about availability of computer code

Data collection	No software was used for data collection
Data analysis	The reads were first processed with 10x Genomics Long Ranger basic. The assembly was then run using 10x Genomics Supernova. ARCS, pipelined with LINKS, was used to pair the Supernova draft assembly sequences Nanopore long reads were used for gap filling via RAILS/Cobbler then the scaffolds were polished with ntEdit. The assembly quality control metrics were calculated using QUAST , BUSCO and CEGMA and a preliminary gene prediction was done via AUGUSTUS.<br>The principal pseudohaplotype annotation was performed using NCBI's in-house Eukaryotic Annotation Pipeline.<br>Previously generated interleaved reads were mapped using the BWA-MEM algorithm and the resulting BAM file was generated via SAMTOOLS view. Deeptools was used to plot the genome coverage. Variants were called using Manta and SNVSniffer. The effect of those called variants were predicted using Galaxy's SNPEff to extract all genes that lost their functions. Those resulting genes were functionally annotated via DAVID. Large-scale structural variants were plotted via Circos. Variant calls statistics were calculated using bcftools stats.<br>BLAST search was run to identify viral genomic insertions

For manuscripts utilizing custom algorithms or software that are central to the research but not yet described in published literature, software must be made available to editors/reviewers. We strongly encourage code deposition in a community repository (e.g. GitHub). See the Nature Research guidelines for submitting code & software for further information.

Data

Policy information about availability of data

All manuscripts must include a data availability statement. This statement should provide the following information, where applicable:

- Accession codes, unique identifiers, or web links for publicly available datasets
- A list of figures that have associated raw data
- A description of any restrictions on data availability

All relevant data supporting the findings of this study are contained within the paper and supplementary files, with the exception of the assembly files, annotation files and RNAseq raw FASTQ sequencing files which are deposited on NCBI database.

Field-specific reporting

Please select the one below that is the best fit for your research. If you are not sure, read the appropriate sections before making your selection.

☒ Life sciences ☐ Behavioural & social sciences ☐ Ecological, evolutionary & environmental sciences

For a reference copy of the document with all sections, see nature.com/documents/nr-reporting-summary-flat.pdf

Life sciences study design

All studies must disclose on these points even when the disclosure is negative.

Sample size	Not applicable.
Data exclusions	Uncharacterized gene IDs were filtered out during gene functional annotation using DAVID.
Replication	Not applicable.
Randomization	Sample allocation was random
Blinding	Given the lack of opportunities for bias in the data collection, we did not consider blinding relevant to the study.

Reporting for specific materials, systems and methods

We require information from authors about some types of materials, experimental systems and methods used in many studies. Here, indicate whether each material, system or method listed is relevant to your study. If you are not sure if a list item applies to your research, read the appropriate section before selecting a response.

Materials & experimental systems

n/a	Involved in the study	
☒	☐	Antibodies
☐	☒	Eukaryotic cell lines
☒	☐	Palaeontology
☒	☐	Animals and other organisms
☒	☐	Human research participants
☒	☐	Clinical data

Methods

n/a	Involved in the study	
☒	☐	ChIP-seq
☒	☐	Flow cytometry
☒	☐	MRI-based neuroimaging

Eukaryotic cell lines

Policy information about cell lines

Cell line source(s)	The Vero cell line was obtained from the Vero WHO master cell bank approved for vaccine production.
Authentication	Cells were authenticated and tested for sterility by WHO.
Mycoplasma contamination	No mycoplasma contamination was reported by WHO.
Commonly misidentified lines (See ICLAC register)	No commonly misidentified cell lines were used in this study.
